# Ovarian reserve in patients with Sjögren’s syndrome: a cross-sectional study

**DOI:** 10.1007/s10067-024-07241-7

**Published:** 2024-11-22

**Authors:** Chiara Mandosi, Viviana Matys, Marianna Deroma, Valentina Del Negro, Lucia Merlino, Marianna Mariani, Roberta Priori, Enrico Ciminello, Emanuela Anastasi, Maria Grazia Porpora, Maria Grazia Piccioni

**Affiliations:** 1 Department of Maternal and Child Health and Urological Sciences, Sapienza University of Rome, Policlinico Umberto I, Viale del Policlinico 155, 00161 Rome, Italy; 2 Rheumatology, Azienda Ospedaliera Universitaria Policlinico Umberto I, Sapienza University of Rome, Viale del, Policlinico 155, Rome, Italy; 3 UniCamillus, Saint Camillus International, University of Health Sciences, Rome, Italy; 4 Scientific Secretariat of the Presidency, Italian National Institute of Health, Rome, Italy; 5 Department of Experimental Medicine, Sapienza University of Rome, Rome, Italy

**Keywords:** Anti-müllerian hormone, Fertility, Ovarian reserve, Sjögren’s syndrome

## Abstract

**Objective:**

This study aimed to assess the potential impact of primary Sjögren’s syndrome (pSS) on fertility and ovarian reserve by evaluating the number of antral ovarian follicles (AFC) through ultrasound and analysing serum levels of anti-müllerian hormone (AMH) and follicle-stimulating hormone (FSH), which are currently the most reliable indicators of fertility potential.

**Method:**

A total of 52 premenopausal women were recruited from the Maternal, Infantile, and Urological Sciences Department at Umberto I Hospital, Sapienza University of Rome. Among them, 26 had pSS, and 26 served as healthy controls. All participants underwent a gynaecological examination, a transvaginal ultrasound, and serum testing for AMH and FSH levels.

**Results:**

The study found that serum AMH levels were significantly lower (*p* = 0.002) in pSS patients compared to the controls, indicating a potential reduction in ovarian reserve in these patients. However, no statistically significant differences were observed in FSH levels between the two groups.

**Conclusions:**

The findings suggest that pSS may have a negative impact on ovarian reserve, as evidenced by lower AMH levels in comparison to age-matched controls. AFC and FSH levels, however, were similar to those of healthy women. These results provide new insights that could be beneficial for this patient population, though further, larger-scale studies are necessary to more comprehensively understand the relationship between pSS and female fertility.

**Table:** 

**Key Points** • *The study assesses the possible impact of pSS on fertility and ovarian reserve by evaluation of AMH, FSH, and AFC.* • *Family planning and fertility are important issues for patients with rheumatic disorders and must be considered and discussed with the patient already at the time of diagnosis, and appropriate counselling must be performed.*

## Introduction 

Primary or isolated Sjögren’s syndrome (pSS) is a chronic autoimmune disease mainly affecting exocrine glands with a typical focal lymphocytic infiltration and associated sicca symptoms. Even if glandular manifestations are the most prevalent, systemic features, such as fatigue and chronic pain [1], and extra-glandular involvement are frequent and may be life-threatening [2, 3]. As in other autoimmune diseases, pSS is more frequent among women compared to men with a ratio of 20:1 [4]. It is estimated that in 14% of cases, the diagnosis of pSS is made before 35 years while peri- and post-menopausal women are more frequently affected [5]. However, at least in industrialized countries, the age of pregnancy may be delayed; therefore, pregnancy-related issues have gained importance also for pSS patients. Despite the female preponderance and the great impact that gynaecological, obstetric, and sexual aspects of the disease can have on the overall quality of life, only a few studies have addressed these points which have been largely overlooked [6–10].

Emerging evidence suggests that autoimmune conditions like pSS may influence ovarian reserve, potentially through mechanisms involving chronic inflammation and the production of proinflammatory cytokines. Understanding the relationship between pSS and ovarian reserve is crucial, as it could have significant implications for fertility management and overall reproductive health in women affected by this condition.

As far as fertility and parity, no significant differences have been reported between pSS and healthy women [11, 12]. Karakus et al. evaluated ovarian reserve in pSS patients demonstrating a reduction in comparison to controls which, according to the authors, could be considered an early sign of premature ovarian insufficiency (POI) [13].

A possible pathogenetic linkage between pSS and impaired ovarian function is the proinflammatory milieu and an excessive production of proinflammatory cytokines which can negatively impact on the ovarian reserve. In fact, cytokines seem to have a possible role in the age-related process of impairment of ovarian reserve, due to the expression of inflammatory genes and promoting apoptotic pathways [14–20].

In addition, some typical symptoms of pSS such as vaginal dryness and dyspareunia may interfere with sexual function and consequently with the possibility to get pregnant, due to the decreased predisposition to have sexual intercourse [6–10, 21]. The management of dyspareunia in women with Sjögren’s syndrome requires a multidisciplinary approach that addresses both the underlying autoimmune condition and the specific symptoms of vaginal dryness. This can include the use of lubricants, moisturizers, and, in some cases, hormone therapy to alleviate vaginal dryness and improve sexual comfort. Moreover, addressing the psychological and emotional aspects of living with a chronic illness is also important, as these can further impact sexual function and overall quality of life.

Our study aimed to investigate the possible effect of pSS on fertility and ovarian reserve through ultrasound evaluation of the number of antral ovarian follicles (AFC) and hormonal assessment of serum anti-müllerian hormone (AMH) and follicle-stimulating hormone levels (FSH), which are currently considered the best methods to assess fertility potential [22–24].

## Materials and methods

### Study design

This is a cross-sectional single-centre study performed at the Maternal, Infantile, and Urological Sciences Department of the Umberto I Hospital, Sapienza University of Rome. Overall, 52 premenopausal women were enrolled in the study from December 2021 to December 2023, after signing a mandatory informed written consent. They were then allocated in two groups of the same size. The study has been approved by the local ethical committee (Sapienza University of Rome, approval number 0000/2021, approval date 26/11/2021). The study was performed in accordance with the ethical standards as laid down in the 1964 Declaration of Helsinki and its later amendments or comparable ethical standards. A written informed consent was obtained from all subjects.

### Cases

Premenopausal patients followed in the outpatient clinic dedicated to the diagnosis and treatment of SS were consecutively enrolled. All patients were classified as pSS according to the American-European Consensus group criteria [25] in the absence of a known overlapping autoimmune disease. Disease duration and the Sjögren's Syndrome Disease Damage Index (SSDDI) were considered [26].

### Controls

As controls women were consecutively enrolled during routine gynaecological consultation. All controls were premenopausal, with sexually active life and with no rheumatologic diseases.

A detailed medical history was collected in all subjects including demographic information, comorbidities, and usual drug intake. Gynaecological and obstetrical medical history was taken with special regard to previous pregnancies or pregnancy investigations. Premature ovarian insufficiency (POI), defined by triads of amenorrhea for at least three cycles or 6 months and elevated FSH serum concentrations over 40 IU/L in two samples at least 1 month apart in women under 40 years of age [27], was excluded in both cases and controls.

### Gynaecological examination

All the subjects enrolled were referred to the Maternal, Infantile, and Urological Sciences Department of Policlinico Umberto I to undergo a gynaecological evaluation. First, vaginal pH was assessed by a simple test, since in premenopausal women the vaginal pH is considered normal when < 4,5 [28]. Then, a regular gynaecological examination was performed, with particular attention to vaginal trophism (presence or absence of dryness, lack of lubrication) and consequently the presence of discomfort (irritation, burning, or pain) during the pelvic examination. Transvaginal ultrasound was performed with a high-frequency transducer (5–9 MHz), to evaluate the uterus and adnexa and the AFC.

### Ovarian reserve

The ovarian reserve was assessed by serum measurement of AMH and FSH, and by sonographic AFC carried out by the same gynaecologist blinded to the medical data. In particular, AFC consists of the sum of antral follicles in both ovaries evaluated in the early follicular phase (day 2–4) of the menstrual cycle. The antral follicle is a follicle with a mean diameter < 10 mm on the 2D US (Tal 2017). Ovarian volume was measured using the equation of an ellipsoid (0.526 × length × width × height) and expressed in cubic centimetres.

Several studies showed that AFC and AMH had the best sensitivity and specificity and consequently the best predictive value for ovarian response to stimulation, so they are useful to define a good ovarian reserve. In particular for AMH, the best cutoff values reported are in the range from 0.5 to 1.1 ng/ml, whereas for AFC the values may range from less than 5 to less than 7 [29–31].

According to the ESHRE consensus [32] at least two of the following three features must be present to define a poor ovarian response (POR) to ovarian stimulation:

- Advanced maternal age (≥ 40 years) or any other risk factor for POR;
- A previous POR (≤ 3 oocytes with a conventional stimulation protocol);
- An abnormal ovarian reserve test (i.e. AFC, five to seven follicles or AMH, 0.5–1.1 ng/ml).

### Blood tests

Blood samples were taken from all patients to determine their hormonal status. Blood collection was performed following a standard protocol. Samples were collected in a red top Vacutainer (Becton, Dickinson and Company, Plymouth, UK), clotted for 60–90 min and centrifuged for 10 min at 1300 × g at room temperature. The serum fractions obtained were subsequently aliquoted in 1.5 ml Eppendorf tubes (Eppendorf srl, Milano, Italy) and frozen at − 80 °C until testing. AMH and FSH serum values were measured by the fully automated system Lumipulse G1200 (Fujirebio-Europe, Gent, Belgium), a chemiluminescence enzyme immunoassay (CLEIA) using a two-step sandwich in immunoreaction cartridges. AMH concentration was determined by Lumipulse® G AMH assay, which displays a detection range comprised between 0.01 and 25 ng/ml, while FSH was measured by Lumipulse® G FSH-N assay which is characterized by a detection range comprised between 0.1 and 250 IU/ml. Both assays had an imprecision (CV %) lower than 10% based on the 95% confidence interval, as declared by the manufacturer.

### Statistical analysis

The statistical analysis was performed by the software R, version 3.6.3 (2020–02–29) “Holding the Windsock”.

The main outcome of the analysis was finding out whether the AMH and FSH scores were significantly different on average between SS and control groups. Such difference was investigated via the Mann–Whitney test for non-paired samples. The significance threshold was fixed equal to *p* < 0.05.

The homogeneity of the patients and the control groups concerning possible confounders was checked by the Welch test for continuous variables and by the chi-square test for categorical variables. Results are reported in terms of “mean” ± “standard deviation (SD)” for continuous variables and absolute frequencies with percentages for categorical variables.

The correlations between disease duration and SSDDI with hormonal status and ovarian reserve for the pSS group were measured by the Pearson correlation coefficient and tested by the Pearson correlation test.

## Results

Twenty-six with pSS (disease duration 55.04 ± 38.84 months) and 26 controls were enrolled. There was no statistically significant difference between the two groups regarding age (*p* = 0.26) and hormonal status (all patients in both groups were premenopausal). BMI, marital status, social status and parity, number of full-term pregnancies, and miscarriages were homogeneous among cases and controls. In both groups, no patients were taking hormonal contraceptives. Table 1 reports the demographic and laboratory data of the patients in the two groups, expressed by mean ± SD for variables of interest.

**Table 1:** Demographic, clinical, and laboratory characteristics in SS and control group

Variables	SS (*n *= 26) Mean ± SD or *n* (%)	Controls (*n *= 26) Mean ± SD or *n* (%)	*p *value
**Demographic data**			
Age (years)	36.84 ± 6.48	34.96 ± 5.06	0.16
BMI (kg/m^2^)	21.4 ± 3.72	20.72 ± 2.09	0.78
Smokers (*n*)	7 (26,9%)	0 (0%)	**0.01**
Duration of menstrual period (days)	6.48 ± 3.2	6.64 ± 3.86	0.79
**Pregnancy history**			
Gravidity (*n*)	1.4 ± 1.58	0.68 ± 1.07	0.09
Parity (*n*)	0.88 ± 0.93	0.48 ± 0.71	0.11
Abortions (*n*)	0.52 ± 0.87	0.2 ± 0.5	0.16
**Gynaecological examination**			
Vaginal dryness	5 (19.2%)	0 (0%)	0.06
Vaginal pH	4.76 ± 0.97	4.56 ± 0.51	0.55
Altered vaginal pH (*n*)	15 (51.72%)	14 (48.28%)	1
**Ultrasound examination**			
Right ovarian AFC (*n*)	3.84 ± 3.36	4.48 ± 3.04	0.36
Left ovarian AFC (*n*)	4.12 ± 3.61	3.56 ± 2.48	0.87
Right ovarian volume (cm^3^)	4.34 ± 2.63	4.83 ± 1.23	**0.04**
Left ovarian volume (cm^3^)	4.05 ± 2.69	4.33 ± 1.36	0.13
**Hormonal status**			
AMH (ng/ml)	1.90 ± 2.05	4.00 ± 2.52	** < 0.01**
FSH (IU/ml)	8.74 ± 9.58	6.87 ± 8.41	0.10

*BMI* body mass index, *AMH* anti-müllerian hormone, *FSH* follicle-stimulating hormone

### Gynaecological examination

All the gynaecological examinations and ultrasound were performed within the 13th day of the menstrual cycle, except for two patients (one case and one control) that were examined on the 17th day of the menstrual period.

All patients participating had a recent Pap Smear with no abnormalities. Vaginal dryness was found only in patients with pSS (5/26, 19.23% within the group) and absent in controls (*p* = 0.06). Vaginal pH was altered (> 4.5) in 15 pSS patients (57.69% within the group) and in 14 controls (53.85% within the group) which was not statistically significant.

### Ultrasound examination

AFC was performed in all patients undergoing ultrasound examination. There were no statistically significant differences between the two groups in right (*p* = 0.36) and left (*p* = 0.87) AFC and left (*p* = 0.13) ovarian volume. The only statistically significant difference was found in the right ovarian volume with 4.34 ± 2.63 cm^3^ for the pSS group and 4.83 ± 1.23 cm^3^ in the controls (*p* = 0.04).

### Hormonal status

AMH and FSH were measured in both groups. The Welch test showed a statistically significant difference in AMH value between the two groups (*p* < 0.01), while no differences were found in FSH values (*p* = 0.10). Serum AMH value in pSS patients was on average significantly lower than in controls with mean values equal to 1.90 ng/ml ± 2.05 and 3.99 ng/ml ± 2.52 respectively, suggesting a possible poorer ovarian reserve in pSS patients. On the contrary, no statistically significant difference was found in FSH values, with a mean FSH in pSS patients: 8.74 IU/ml ± 9.58 vs a mean FSH in the control group: 6.87 IU/ml ± 8.41 (*p* value = 0.10).

Furthermore, in pSS patients, no correlation was found between disease duration and SSDDI and hormonal status and ovarian reserve.

## Discussion

In this study, 26 women with pSS and 26 healthy controls were enrolled to compare various factors related to ovarian reserve. Both groups were homogeneous in their characteristics.

Key findings include:

- Vaginal dryness was observed in 19.23% of pSS patients but not in controls.
- AMH levels were significantly lower in pSS patients (1.90 ng/mL ± 2.05) compared to controls (3.99 ng/mL ± 2.52, *p* < 0.01), indicating a potential reduction in ovarian reserve.
- FSH levels did not differ significantly between the groups (*p* = 0.10).
- No correlation was found between disease duration and ovarian reserve in pSS patients.

Even if pSS is generally diagnosed after menopause, fertility and ovarian reserve are two aspects of relevant importance for women in their childbearing age with this disorder.

To date, only one preliminary study [13] evaluated the ovarian reserve analysing AMH and AFC, as well as other hormones such as LH, FSH, estradiol, and prolactin, in a group of pSS patients in comparison with healthy controls. The authors reported lower values of AMH and AFC in patients with pSS, suggesting that this result may represent an early sign of premature ovarian failure [13]. This feature also characterizes other autoimmune rheumatic diseases such as rheumatoid arthritis (RA) or Systemic Lupus Erythematosus (SLE) where systemic inflammation is relevant. The demonstration of a better ovarian response to gonadotropins in IL-1 beta-deficient mice [20] and the negative correlation between serum AMH level and IL-6 [19] support the view that inflammation has a negative impact on the ovarian reserve [33, 34].

In this study, ovarian reserve has been investigated through the measurement of the serum level of AMH, the evaluation of the AFC and ovarian volume (OV), which currently are considered the best screening tools to assess women’s fertility [35]. Also, serum FSH considered less accurate because of its instability level through the menstrual cycle [36–38] was measured. Serum AMH is secreted by the granulosa cells of the early follicle and is considered an important marker of ovarian failure. Its level is proportional to the number of developing follicles, and it is strictly associated with the follicle pool [23]. Reduced AMH levels can be detected in women using hormonal contraceptives [35], but none of the healthy or diseased women included in the present study were taking exogenous hormones for at least 1 year before enrollment. Also, BMI, which is known to have an impact on AMH level [39] was comparable between pSS and control subjects. Even if in the pSS group the number of smokers is higher than in the control group, a recent prospective cross-sectional study did not demonstrate any significant difference in quantitative ovarian reserve markers, in terms of AMH serum level, between current smokers, ex-smokers, and never-smokers [40]. Therefore, the observation of a significantly reduced serum concentration of AMH in pSS compared to control seems to be a feature of ovarian dysfunction in pSS patients during childbearing age as previously suggested by Karakus and colleagues [13].

However, no statistically significant differences between the two groups were found in ovarian volume, FSH levels, and the AFC, which is known to present some potential limitations due to hardware and operator variability and the absence of defined category criteria [22].

Our results are in line with those obtained in women with polymyositis (PM) where serum AMH resulted significantly lower in comparison to controls while the number of the AFC was similar in both groups [41].

Moreover, no statistically significant difference was found between the two groups regarding the number of previous pregnancies or duration of menstrual cycles, so only subclinical abnormalities in the ovarian reserve have been detected, possibly suggesting the risk of premature ovarian failure in women with pSS.

In fact, other studies have previously failed to demonstrate any difference regarding menstrual function, fertility, and parity in women with pSS in comparison to healthy women [6, 7, 9, 12, 42].

Finally, in a very recent study, Mao et al. investigated fertility and pregnancy outcomes in patients with primary Sjögren syndrome. In this multi-central retrospective cohort study, they found that ovarian reserve including AMH and AFC were significantly lower in the pSS group vs comparison (0.8 vs. 2.9 ng/mL, *P* < 0.001; 6.0 vs. 10.0, *P* < 0.001, respectively). Furthermore, they discovered that patients with pSS exhibit worse conditions in terms of deteriorated oocyte and embryo development in vitro fertilization (IVF) cycles [43].

Our results are in line with this recent study, reinforcing the hypothesis that there is a possible link between fertility and pSS and that this disease can worsen oocyte quality and ovarian reserve due to several possible factors. However, more extensive studies will have to be conducted to confirm this data and to find possible pathogenic hypotheses linking infertility with this rheumatic pathology.

This study has some limitations. The main limitation is the small number of participants, but, since pSS is a rare condition among women in their childbearing age, our findings offer potentially useful information for this patient population.

Furthermore, patients were not classified into age classes but were made up of a single group, and this could be a possible confounding factor regarding the AMH values, even though all patients were in pre-menopausal age.

Moreover, we did not consider other possible confounding factors such as environmental or lifestyle factors.

Finally, we could not take into consideration every possible lifestyle factor, and the observational nature of this design leaves the possibility of residual confounding. Long-term follow-up studies are needed to verify whether the mild abnormalities found in ovarian reserve predict an upcoming insufficiency.

## Conclusions

The impact of autoimmune diseases on fertility and premature ovarian failure (POF) remains a subject of ongoing debate in the literature, with limited studies addressing this complex issue. In this study, we explored the potential effect of primary Sjögren’s syndrome on ovarian reserve, revealing that patients with pSS had significantly lower levels of AMH compared to age-matched controls. However, other indicators of ovarian reserve, such as ovarian volume, AFC, and FSH levels, were similar to those of healthy women, making it premature to conclude that pSS patients are at increased risk of POF. Nevertheless, a prolonged follow-up is essential to determine whether the observed low AMH levels might be a predictor of POF in women with pSS.

## Data Availability

The data supporting this study's findings are available from the corresponding author, [V.M.], upon reasonable request.
